# Expression of ESE-3 Isoforms in Immunogenic and Tolerogenic Human Monocyte-Derived Dendritic Cells

**DOI:** 10.1371/journal.pone.0049577

**Published:** 2012-11-19

**Authors:** Florian Sprater, Arnt-Ove Hovden, Silke Appel

**Affiliations:** Broegelmann Research Laboratory, The Gade Institute, University of Bergen, Bergen, Norway; Copenhagen University Hospital at Herlev, Denmark

## Abstract

Dendritic cells (DC) are the only hematopoietic cells expressing the epithelial specific Ets transcription factor ESE-3. Here we analyzed presence and quantity of isoforms ESE-3a, ESE-3b and ESE-3j in various immunogenic and tolerogenic human monocyte-derived DC (moDC) and blood DC populations using quantitative real time PCR and immunoblot analyses. ESE-3a and ESE-3b were detectable in all moDC populations with ESE-3b being the main transcript. ESE-3b expression was upregulated in immunogenic moDC and downregulated in tolerogenic moDC compared to immature moDC. ESE-3a had similar transcript levels in immature and immunogenic moDC and had very low levels in tolerogenic moDC. In blood DC populations only splice variant ESE-3b was detectable. ESE-3j was not detectable in any of the DC populations. These findings suggest that ESE-3b is the functionally most important ESE-3 isoform in DC.

## Introduction

Dendritic cells (DC) are the most potent antigen presenting cells with the unique ability to induce and maintain primary immune responses [1]. In the periphery, DC acquire antigens and migrate to the lymph nodes where peptide antigens are presented to T lymphocytes. During this pathway, immature DC, capable of antigen uptake, differentiate into mature DC. Mature DC are characterized by high surface expression of MHC class II and co-stimulatory molecules able to induce an immune response. Furthermore, DC play an important role in tolerance induction [2] as immature DC within peripheral tissues continuously sample the surroundings for antigens, e.g. from apoptotic cells. In the absence of maturation stimuli, DC present self-peptide-MHC complexes to circulating naive T cells in peripheral lymphoid organs. In the case of autoreactivity, the T cells will be deleted or become anergic, thereby avoiding autoimmunity [3]–[5].

Due to the outstanding capacity of DC to process and present antigenic peptides to T lymphocytes, several concepts have been developed to use DC in cancer immunotherapy. The basic idea is to generate sufficient amounts of immunogenic DC from progenitor cells *ex vivo*, load them with tumor specific antigens and re-inject them into the patient where they are then supposed to initiate an immune response towards the tumor cells [6], [7]. Though the theory behind it is very logical, these therapeutic approaches have only shown modest results in the clinic so far [8], [9]. The choice of the DC population has been suggested to be very important in the success of the vaccine [10], [11]. Moreover, during the past few years, the possibility to utilize tolerogenic DC-based vaccines as a clinical modality to treat autoimmune diseases has been investigated with promising results in mouse models [12]–[14].

We have previously reported that the transcription factor ESE-3 is involved in the development of monocyte-derived DC (moDC) [15]. ESE-3 (also known as EHF) belongs to the family of E26 transformation specific (Ets) transcription factors. Members of the Ets family are characterized by a conserved winged helix-turn-helix DNA binding domain called Ets domain enabling them to bind the core DNA sequence 5′-GGA(A/T)-3′ [16]. Several members of the Ets family are proto-oncogenes involved in cancer development and tumor invasion [17], [18]. Others play crucial roles in the proliferation as well as in differentiation of epithelial and hematopoietic cells [19], [20]. To name a few, it was shown that PU.1 and Ets1 are important in T cell development [21]. Regarding DC, PU.1 is required for development of myeloid-derived but not for lymphoid-derived DC [22]. Moreover, Spi-B expression seems to be crucial during development of human plasmacytoid DC [23].

ESE-3 [24] belongs to the epithelial specific subset of Ets transcription factors, including ESE-1 (also known as ELF-3) [25] and ESE-2 (also known as ELF-5) [26]. ESE-1 and ESE-3 have been shown to be involved in the regulation of allergic airway inflammation [27], [28]. Furthermore, a genome-wide association study has found linkage of cystic fibrosis lung disease severity with a locus near the ESE-3 gene on chromosome 11p13 [29], suggesting that ESE-3 might be involved in regulating cell differentiation in the airways. Moreover, it was recently described that DC from ESE-1^−/−^ mice produce reduced amounts of IL-6 resulting in impaired Th17 differentiation in these animals [28].

**Figure 1:**
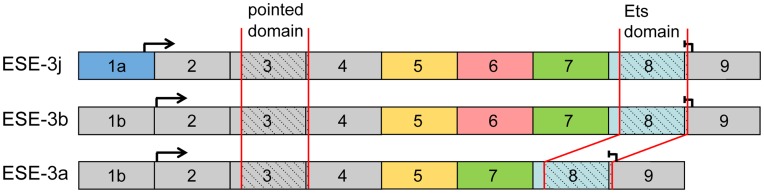
Exon composition of ESE-3 transcript variants. Exons are marked with numbers 1–9. Critical exons for distinction between the splice variants are color-coded. The different domains and translational start (→) and stop (|<$>\vskip 1pt\raster="rg1"<$>) are marked. An alternative exon 1a distinguishes ESE-3j from ESE-3a and ESE-3b. Translation of ESE-3j is initiated in exon 1a, whereas translation of ESE-3a and ESE-3b starts in exon 2. ESE-3a is the shortest transcript variant owing to the lack of exon 6.

Epithelial cells have important functions in balancing immune responses and immune tolerance. Different pathways are involved in promoting changes in cytokine and chemokine secretion by epithelial cells as well as in the display of molecules on the surface of epithelial cells [30]. Interestingly, ESE-3 expression in bronchial epithelial cells is upregulated by the inflammatory cytokines IL-1β and TNF-α. Since ESE-3 expression was shown in epithelial as well as in dendritic cells [15], [31] it is tempting to link the role of ESE-3 directly to the immunoregulating functions of these two cell types. However, the role of ESE-3 in the transcriptional networks in these cells has yet to be assessed.

ESE-3 is a transcriptional repressor of several genes that are positively regulated by MAP kinase signalling [31]. ESE-3 requires high affinity binding sites with the suggested consensus sequence 5′-AGGAAGT-3′ [31]. Moreover, downregulation of ESE-3 has been described to be involved in prostate tumorigenesis [32], [33]. Albino and colleagues recently showed that loss of ESE-3 induces epithelial-to-mesenchymal transition in prostate epithelial cells, suggesting ESE-3 to be a key regulator in the development of prostate cancer [33]. On the other hand, overexpression of ESE-3 was reported in ovarian cancer [34] and has therefore been proposed as a predictive marker for poor survival of patients [35]. It has also been suggested that survival of colon tumor cells containing wild-type p53 are dependent on overexpression of ESE-3 [36].

The function of ESE-3 during the development and maturation of DC is still elusive. Knockdown experiments using siRNA suggest that ESE-3 is crucial during moDC development. In addition, it was shown that DC are the only hematopoietic cells that express this transcription factor [15].

**Figure 2:**
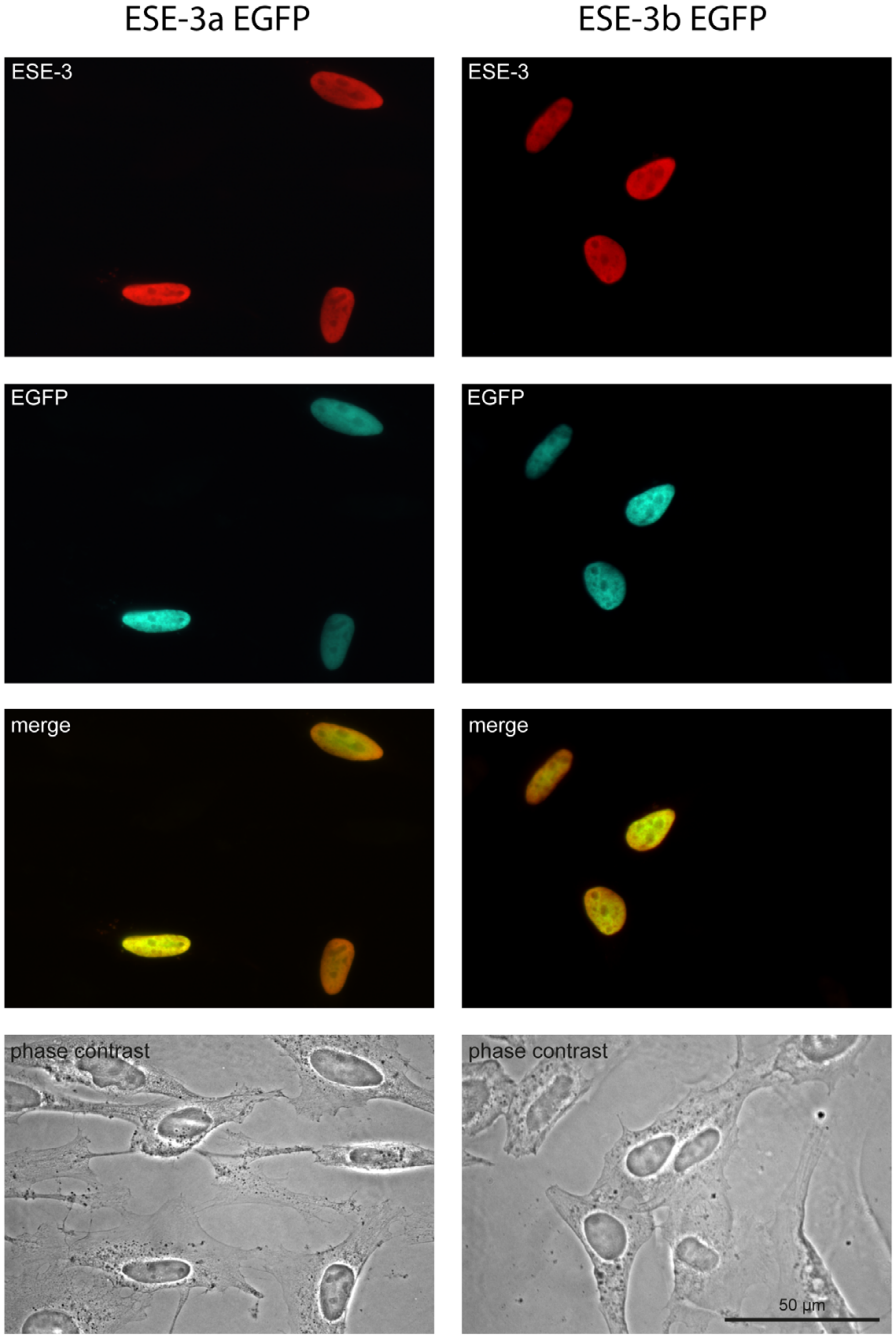
Overexpressed ESE-3a and ESE-3b are located in the nucleus of HeLa cells. HeLa cells were transfected with expression vectors coding for ESE-3a-EGFP and ESE-3b-EGFP. After 24 h, cells were fixed and subjected to immunocytochemistry using a monoclonal antibody specific for human ESE-3. The ESE-3a- and ESE-3b-constructs localized exclusively to the nucleus as revealed by both the intrinsic green fluorescence and the ESE-3 immunoreactivity. One representative experiment out of three is shown. Scale bar: 50 µM.

**Figure 3:**
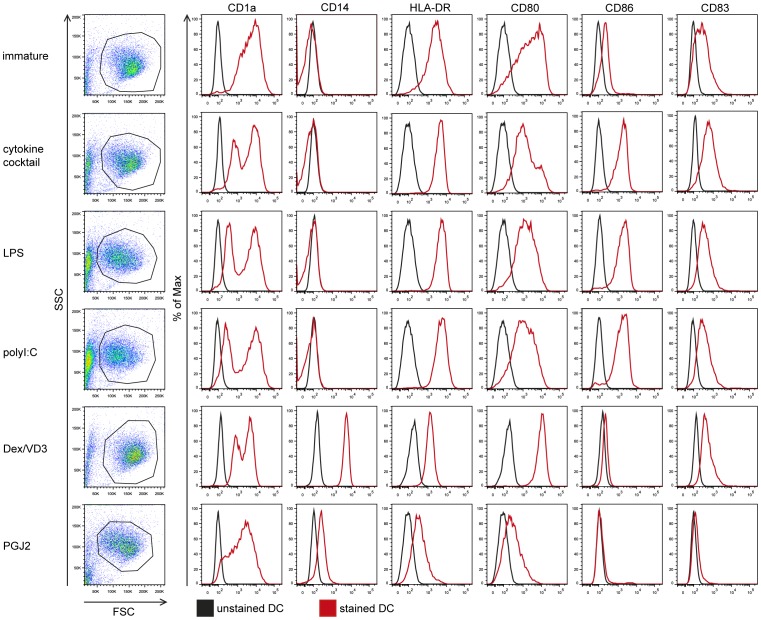
Phenotypic characterization of immunogenic and tolerogenic moDC populations by flow cytometry. Monocytes were negatively selected from PBMC using magnetic beads. Immature moDC were generated with IL-4 and GM-CSF for 6 days. 15d-PGJ_2_ (PGJ2 DC) and dexamethasone plus 1α,25-dihydroxyvitamin were added to generate tolerogenic moDC, respectively (PGJ2 DC and Dex/VD3 DC). To generate immunogenic moDC, immature moDC were stimulated for 24 h with LPS, polyI:C and a cytokine cocktail containing TNF-α, IL-1β, IL-6 and PGE_2_, respectively. The phenotypes of the cells were analyzed by flow cytometry. Live cells were gated according to FSC/SSC. One representative experiment out of three is shown.

**Figure 4:**
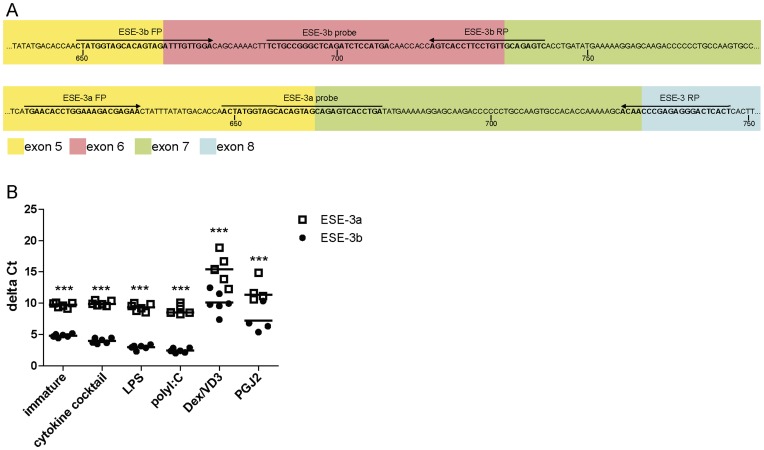
ESE-3b is the main ESE-3 isoform in moDC. Monocytes were negatively selected from PBMC using magnetic beads. Immature moDC were generated with IL-4 and GM-CSF for 6 days. 15d-PGJ_2_ (PGJ2 DC) and dexamethasone plus 1α,25-dihydroxyvitamin were added to generate tolerogenic moDC, respectively (PGJ2 DC and Dex/VD3 DC). To generate immunogenic moDC, immature moDC were stimulated for 24 h with LPS, polyI:C and a cytokine cocktail containing TNF-α, IL-1β, IL-6 and PGE_2_, respectively. ESE-3a and ESE-3b mRNA levels were determined by quantitative RT-PCR analyses. (A) Primers and probes used for quantitative real-time RT PCR are marked. Exons are color-coded as in Figure 1. Positions of the base pairs of the open reading frames are indicated by numbers. (B) Relative amounts of ESE-3a and ESE-3b cDNA in various moDC populations. Transcript levels of GAPDH were used to calculate delta Ct values. Lower delta Ct values indicate higher amounts of target mRNA. N = 4–6. Significant differences between delta Ct values of ESE-3a and ESE-3b within the same moDC population are indicated. ***p<0.001.

Not much is known about the different isoforms of ESE-3. Until now, three different transcript variants have been identified (Figure 1). They encode three different isoforms, ESE-3a (GenBank acc.-no. NM_001206615), ESE-3b (GenBank acc.-no. NM_012153) and ESE-3j (GenBank acc.-no. NM_001206616). ESE-3j is the longest transcript with an alternative exon 1 and start codon as ESE-3a and ESE-3b. ESE-3a is the shortest transcript lacking exon 6 which encodes 23 amino acids. Sequence analyses disclosed an open reading frame encoding 277 amino acids with a calculated molecular weight of 32 kDa for ESE-3a, 300 amino acids with a calculated molecular weight of 35 kDa for ESE-3b [24] and 322 amino acids with a calculated molecular weight of 37 kDa for ESE-3j. Computer based analyses failed to predict any functional properties for the domain that is removed in ESE-3a. Exons 2 and 3 encode a pointed domain responsible for protein-protein interactions, exons 8 and 9 encode the DNA-binding Ets domain constituting the winged helix-turn-helix motif (Figure 1).

**Figure 5:**
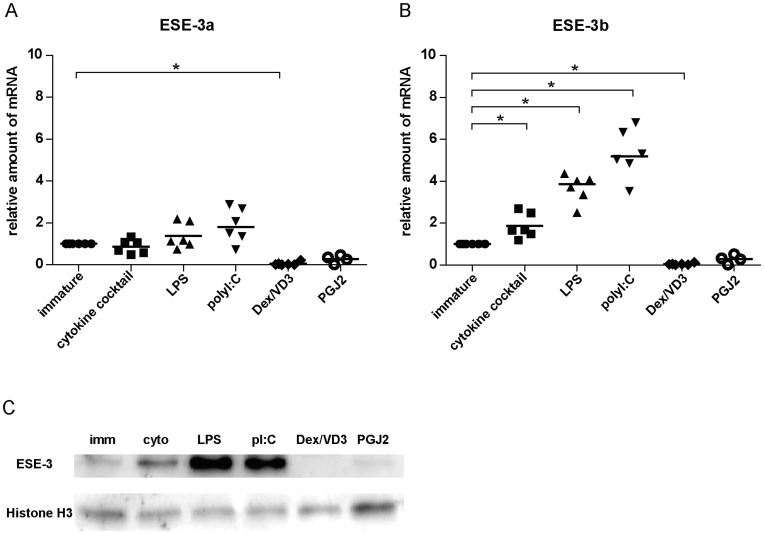
ESE-3b expression is increased in immunogenic moDC. Monocytes were negatively selected from PBMC using magnetic beads. Immature moDC were generated with IL-4 and GM-CSF for 6 days. 15d-PGJ_2_ (PGJ2 DC) and dexamethasone plus 1α, 25-dihydroxyvitamin were added to generate tolerogenic moDC, respectively (PGJ2 DC and Dex/VD3 DC). To generate immunogenic moDC, immature moDC were stimulated for 24 h with LPS, polyI:C and a cytokine cocktail containing TNF-α, IL-1β, IL-6 and PGE_2_, respectively. (A) ESE3a and (B) ESE-3b mRNA levels were analyzed by quantitative real-time RT PCR using isoform specific primer/probes. GAPDH was used for normalization, immature moDC were used as reference. N = 4–6, *p<0.05. (C) Protein levels of ESE-3 in nuclear extracts were analyzed by Western blotting using a monoclonal antibody specific for human ESE-3. Histone H3 was included to ensure equal loading of the gel. One representative experiment out of three is shown.

**Figure 6:**
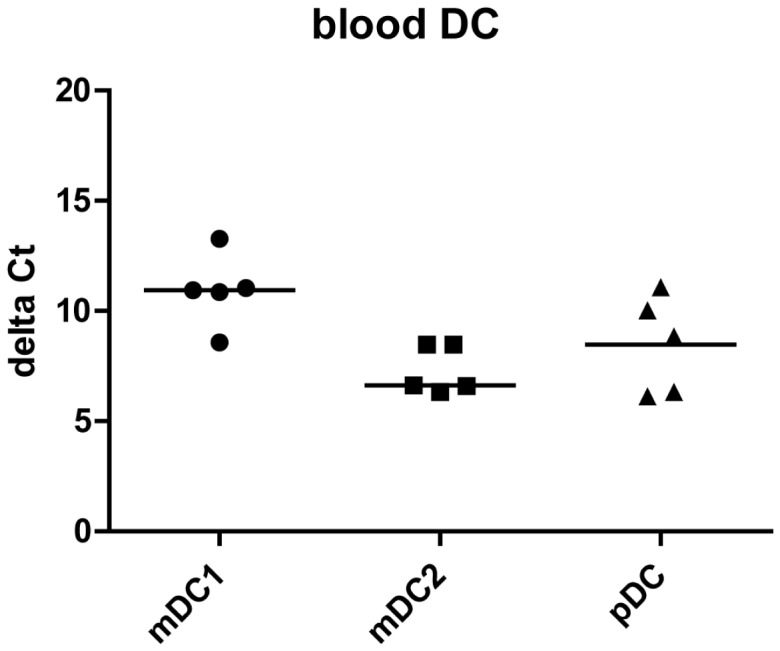
ESE-3b is the only ESE-3 isoform transcribed in blood DC. MDC1, mDC2 and pDC were isolated from PBMC by positive selection with magnetic beads. Quantitative real-time RT-PCR using isoform specific primer/probes was used to analyze mRNA levels of ESE-3a and ESE-3b. GAPDH was used for normalization. No signal for ESE-3a was detected. N = 5.

For improvement of DC based immunotherapy it is crucial to get a better understanding of the molecular mechanism involved in DC development and maturation. Here, we demonstrate that ESE-3a and ESE-3b isoforms are present in various moDC populations with full-length ESE-3b being the main isoform also present in blood DC populations. ESE-3j was not detected in any DC population analyzed. ESE-3b expression was upregulated in immunogenic moDC and downregulated in tolerogenic moDC suggesting an important function in immunogenic DC.

## Materials and Methods

### Generation of Monocyte Derived DC

Buffy coat preparations from healthy blood donors (Blood Bank, Haukeland University Hospital, Bergen, Norway) were used as a source for monocytes as described previously [37]. Briefly, mononuclear cells from peripheral blood were isolated by density gradient centrifugation followed by a negative selection of monocytes using the Dynabeads Untouched Human Monocytes Kit (Invitrogen, Carlsbad, CA) following the manufacturer’s instructions. For the generation of immature moDC, monocytes were cultured in RP10 medium [RPMI 1640 (Cambrex Bioscience, Verviers, Belgium), supplemented with 10% FCS (PAA, Pasching, Austria), 100 units/ml penicillin and 100 mg/ml streptomycin (Sigma-Aldrich, St Louis, MO), IL-4 (20 ng/ml; ImmunoTools, Friesoythe, Germany) and GM-CSF (100 ng/ml; ImmunoTools)] for 6 days. The cytokines were replenished every 2–3 days. To generate immunogenic DC, immature DC were stimulated with LPS (1 µg/ml; Sigma-Aldrich), polyI:C (50 µg/ml; Sigma-Aldrich), and with a cytokine cocktail containing TNF-α (10 ng/ml; ImmunoTools), IL-1β (10 ng/ml; ImmunoTools), IL-6 (1000 U/ml; ImmunoTools) and PGE_2_ (1 µg/ml; Sigma-Aldrich), respectively, for 24 h. For the generation of two tolerogenic DC populations, IL-4, GM-CSF and 15d-PGJ_2_ (5 µM; Biomol, Hamburg, Germany) were added to one population of monocytes from day 0 and replenished every 2–3 days (PGJ2-DC), and IL-4, GM-CSF (from day 0), dexamethasone (day 3 and 6; 10^−6^ M; Sigma-Aldrich) and 1α,25-dihydroxyvitamin D3 (day 6; 10^−10^ M; Sigma-Aldrich) were added to another cell population (Dex/VD3 DC).

### Isolation of DC From Peripheral Blood

Peripheral blood DC populations [type-1 myeloid DC (mDC1), type-2 myeloid DC (mDC2) and plasmacytoid DC (pDC)] were isolated from buffy coat preparations from healthy donors (Blood Bank, Haukeland University Hospital) using magnetic cell sorting (MACS) technology according to the manufacturer’s protocols (CD1c Dendritic Cell Isolation Kit, CD141 MicroBead Kit, CD304 MicroBead Kit, all from Miltenyi Biotec, Bergisch Gladbach, Germany).

### RNA Isolation, cDNA Synthesis, PCR and Real-time RT-PCR

Total RNA was isolated using the RNeasy Mini or RNeasy Plus Mini Kit (QIAGEN, Hilden, Germany) following the manufacturer’s protocol. Up to 2 µg total RNA was used in a 20 µl cDNA synthesis reaction using RevertAid Reverse Transcriptase (Fermentas, St. Leon-Rot, Germany) according to the manufacturer’s recommendations. Two reactions were performed for each sample using Oligo(dT)_18_ primer and random hexamer primer. The two reactions were pooled afterwards. For analyzing ESE-3j expression, a PCR with 35 cycles was performed using the primers ESE-3j fwd: 5′- GTTGCCGGAGAGAAGAGGAT and ESE-3j rev: 5′- GGTCCAGTACTGAGGATGAAT and approximately 50 ng of cDNA as template. The same amount of cDNA was used to analyze ESE-3a and ESE-3b transcript levels in a quantitative real-time RT-PCR using Taqman technology. For detection an ABI 7500 real time PCR system (Applied Biosystems, Carlsbad, USA) was used. The reactions were run in duplicates. Primer and probes used were as follows: ESE-3a FP: 5′-TGAACACCTGGAAAGACGAGAA, ESE-3a RP: 5′-AGTGAGTCCCTCTCGGGTTGT, ESE-3a probe: 5′-[6FAM]TCATATCAGGTGACTCTGCTACTGTGCTACCATAGT[BHQ1], ESE-3b FP: 5′-CTATGGTAGCACAGTAGATTT GTTGGA, ESE-3b RP: 5′-GACTCTGCAACAGGAAGGTGACT, ESE-3b probe: 5′-[6FAM]TCTGCCGGGCTCAGATCTCCATGA[BHQ1], GAPDH FP: 5′-CCACATCGCT CAGACACCAT, GAPDH RP: 5′-GGCAACAATATCCACTTTACCAGAGT, and GAPDH Probe: 5′-[6FAM]ACCAAATCCGTTGACTCCGACCTTCA[BHQ1]. For each RT-PCR reaction 1×10^4^ copies from ESE-3a pEGFP-N1 and ESE-3b pEGFP-N1was included as control for specificity and efficiency of the primer/probe combination. Transcript levels of GAPDH were used for normalization. To determine the relative expression of the ESE-3 isoforms in the different moDC populations, immature moDC were used as reference.

### Western Blotting

5×10^5^ cells were used to prepare nuclear extracts as described previously [38]. Approximately 20 µg nuclear extract were loaded on each lane of a 12% sodium dodecyl sulfate (SDS)-polyacrylamide gel and transferred to a nitrocellulose membrane (Bio-Rad Laboratories, Hercules, CA). Membranes were stained with Ponceau S solution to confirm equal amounts of protein in each lane. This was verified by using a histone H3 antibody (D1H2, Cell Signalling, Boston, MA) in one experiment. The blot was probed over night at 4°C with a monoclonal rat anti ESE-3 antibody (5A5.5, Lifespan, Seattle, WA) diluted 1∶200. As secondary antibody a HRP-coupled goat anti rat antibody (Santa Cruz Biotechnology, Santa Cruz, CA) was used in a 1∶1000 dilution. Signal West Femto was used as substrate (Pierce, Thermo Fisher Scientific, Rockford, IL). Proteins were visualized with a ChemicDoc XRS system and analyzed with Quantity One software (both from Bio-Rad Laboratories).

### Flowcytometry

Immunostaining was performed to determine the phenotype of the different DC populations. Cells were incubated with FcR-block (Miltenyi Biotec, 2,5 µl/1×10^5^ cells, 10 min, room temperature) followed by incubation with titrated amounts of antibodies (10 min, room temperature). Subsequently the cells were washed and analyzed on a LSRFortessa Cell Analyzer (BD Biosciences, Heidelberg, Germany). The following antibodies were used: CD1a-PE (HI 149, ImmunoTools), CD14-FITC (18D11, ImmunoTools), HLA-DR-APC (HL-39; AbD Serotec, Düsseldorf, Germany), CD86-FITC (BU63; AbD Serotec), CD83-PE (HB15, AbD Serotec), CD80-APC (MEM-233, ImmunoTools). FlowJo software (Tree Star, Ashland, OR) was used for the analysis. In the negative control samples 1% false-positive events were accepted throughout the experiments.

### Cloning of ESE3a and ESE3b into pEGFP-N1

For the full-length isoform ESE-3b, the primers ESE3 fwd: 5′-GGCGGCTAGCCACCATGATTCTGGAAGGAGGTGG/ESE3 rev: 5′-GCGGACCGGTCCGTTTTCATTTTCTCTCCATCC were used with cDNA from moDC as template. NheI/AgeI was used for cloning into pEGFP-N1 (Clontech, Saint-Germain-en-Laye, France).

For the ESE3a isoform two amplicons were generated using the primers ESE3fwd: 5′-GGCGGCTAGCCACCATGATTCTGGAAGGAGGTGG/ESE3rev splice variant: 5′- TACT GTGCTACCATAGTTGG and ESE3fwd splice variant: 5′-GCAGAGTCACCTGATATGAA/ESE3rev: 5′-GCGGACCGGTCCGTTTTCATTTTCTCTCCATCC. The longer fragment (ESE3fwd/ESE3rev splice variant) was phosphorylated and the two fragments were ligated. A new PCR was performed using the ligated fragments as template and the primers ESE3fwd/ESE3rev. NheI/AgeI was used to clone the product into pEGFP-N1. Both constructs, ESE3a-pEGFP-N1 and ESE3b-pEGFP-N1, were transformed into JM109 cells. Positive clones were selected using LB-plates containing kanamycin. Correct clones were confirmed by sequencing.

### Immunocytochemistry

HeLa-S3 cells (DSMZ, Braunschweig, Germany) were cultured in DMEM medium (Gibco, Invitrogen) with 10% FCS (PAA), 100 units/ml penicillin and 100 mg/ml streptomycin (both Sigma-Aldrich). Transfection was performed in a 24 well plate using Effectene Transfection Reagent (QIAGEN, Hilden, Germany) according to the manufacturer’s protocol. After 24 h cells were fixed for 30 minutes with ice cold 4% formaldehyde (Sigma-Aldrich) and permeabilized with 0.5% Triton X-100 (Sigma-Aldrich) for 15 minutes at room temperature. After blocking with 10% FCS (1 h, room temperature), a 1∶200 dilution of rat anti-ESE3 antibody (5A5.5, Lifespan) in 10% FCS was added to the fixed cells and incubated over night at 4°C. For detection, a secondary Texas Red-x goat anti-rat IgG antibody (Invitrogen) was added for 1 h at room temperature in a 1∶1000 dilution. Nuclei were stained using DAPI (0.5 µg/ml).

### Statistical Analyses

All statistical analyses were performed with Prism (GraphPad software Inc., La Jolla, CA). Wilcoxon signed rank test was applied to analyze differences in the median of relative mRNA levels of moDC populations, a one-way analysis of variance (ANOVA) was used to analyze differences in delta Ct values in the different blood DC populations, and a 2-way ANOVA with Bonferroni post-test was utilized to analyze differences in delta Ct values for ESE-3a and ESE-3b expression within each moDC population. A p value<0.05 was considered statistically significant.

## Results

### Overexpressed ESE-3a and ESE-3b is Located in the Nucleus of HeLa Cells

ESE-3 is a transcription factor and should therefore be located in the nucleus of cells when active. However, the DNA-sequences of all ESE-3 splice variants lack a nuclear localization signal. Here we wanted to investigate if changes in the primary structures of the ESE-3 isoforms have an impact on cellular localization. Since there are no isoform-specific ESE-3 antibodies available we transiently expressed C-terminally EGFP-fused ESE-3a and ESE-3b constructs in HeLa cells. One day post transfection both proteins were exclusively localized to the nucleus as revealed by both the intrinsic green fluorescence and by ESE-3 immunocytochemistry (Figure 2). Nuclei were additionally stained with DAPI (data not shown).

### ESE-3a and ESE-3b are Transcribed in Human moDC with ESE-3b being the Main Transcript Upregulated Upon Stimulation of DC

We next wanted to compare transcript levels of ESE-3a, ESE-3b and ESE-3j in various immunogenic and tolerogenic moDC populations. The phenotype of the generated moDC populations was verified by flow cytometry (Figure 3). As expected, moDC matured with the cytokine cocktail, LPS or polyI:C had high expression of CD1a and CD80, upregulation of CD83, CD86 and HLA-DR compared to immature DC, and had lost CD14 expression. Tolerogenic DC generated with Dex/VD3 showed high expression of CD1a, CD80 and HLA-DR. Compared to immature DC they showed a slight increase of CD83 expression but no upregulation of CD86 on their surface. In addition, they still expressed monocyte marker CD14. MoDC generated in the presence of PGJ_2_ had a reduced expression of CD1a, HLA-DR, CD80, CD83 and CD86 compared to immature moDC and were slightly positive for CD14.

For verification of the amplification efficiency of the primer/probe combinations (Figure 4A) we conducted control reactions with plasmid DNA harboring the open reading frames of ESE-3a and ESE-3b, respectively (data not shown). Analyzing transcript levels of the splice variants ESE-3a and ESE-3b showed that ESE-3b was the main transcript in all moDC populations determined by lower delta Ct values compared to ESE-3a (Figure 4B).

ESE-3j could not be detected in any moDC population analyzed by conventional PCR (data not shown).

ESE-3b, but not ESE-3a transcription was significantly upregulated upon stimulation of the cells (Figure 5A, B). Presence of Dex/VD3 and PGJ_2_ during moDC generation resulted in decreased amounts of ESE-3a and ESE-3b mRNA compared to immature moDC (Figure 5A, B). Particular in the DC population treated with Dex/VD3, ESE-3a was hardly detectable (Ct values around 40).

In order to confirm the differential expression of the ESE-3 isoforms on protein level, we analyzed isolated nuclei for ESE-3 expression by Western blot using an antibody recognizing all ESE-3 isoforms. Only one band corresponding to ESE-3b was detectable on the blots (Figure 5C). Strong immunoreactivity of ESE-3 was detectable in nuclei of immunogenic moDC populations with moDC matured with LPS or polyI:C giving the highest signal. Both tolerogenic moDC populations showed almost no ESE-3b expression confirming the real time RT-PCR results.

### ESE-3b is the Only ESE-3 Isoform Transcribed in Blood DC

To investigate if the results obtained from moDC populations were similar for blood DC populations, we isolated mDC1, mDC2 and pDC from buffy coat blood. In all three blood DC populations, only ESE-3b mRNA was detectable with mDC1 showing the lowest mRNA levels indicated by the highest delta Ct values (Figure 6). However, the observed differences were not statistically significant. ESE-3j could not be detected in any blood DC population (data not shown).

## Discussion

We previously reported that the epithelial specific Ets transcription factor ESE-3 is upregulated in moDC stimulated with different compounds whereas IL-10 treatment inhibiting DC development from monocytes also impedes induction of ESE-3 expression [15]. Here we pursued the study and analyzed ESE-3 isoforms ESE-3a, ESE-3b and ESE-3j in various immunogenic and tolerogenic moDC populations and blood DC populations.

We first focused on the cellular localization of the two isoforms ESE-3a and ESE-3b. It was shown previously that ESE-3 is localized in the nucleus [31], but the antibody used did not discriminate between the different isoforms. We therefore transiently expressed EGFP-fused ESE-3a and ESE-3b constructs in HeLa cells. Both isoforms were detectable only in the nuclei of transfected cells (Figure 2). As none of the isoforms contain a nuclear localization signal, the Ets domain itself might be involved in the nuclear localization as shown previously for the Ets transcription factor PU.1 [39]. The exclusive localization of all ESE-3 isoforms in the nucleus suggests that ESE-3 is not directly regulated via cytoplasmic factors inhibiting its nuclear localization as described for Ets factor Elf-1 [40] and the NF-kB family of transcription factors [41].

We then analyzed expression of the different ESE-3 isoforms in various immunogenic and tolerogenic moDC populations to elucidate if different expression patterns of ESE-3 could be linked to the functionally different DC populations. We used TLR4 ligand LPS, TLR3 ligand polyI:C and the Jonuleit cytokine cocktail most commonly used in today’s DC-based tumor vaccines consisting of IL-1β, IL-6, TNF-α and PGE_2_ [42] for the generation of immunogenic DC. Tolerogenic moDC were generated with PGJ_2_ or Dex/VD3. The manipulation of DC with PGJ_2_ *in vitro* has been described as a potential way for the generation of tolerogenic DC [43]. The addition of a combination of dexamethasone and vitamin D3 during DC generation has also been reported to induce tolerogenic DC [12], [44], [45]. In animal models, therapeutic vaccination with these cells has been shown to decrease disease severity and inhibit progression of arthritis [46].

In our experiments, all three immunogenic moDC populations showed significantly increased levels of ESE-3b mRNA compared to immature moDC. In contrast, ESE-3b transcription was downregulated in the two tolerogenic DC populations (Figure 5A, B). Our findings are in line with our previous results and point out the importance of ESE-3 in immunogenic DC development. However, in the previous study we did not differentiate between ESE-3a and ESE-3b mRNA levels. Since upregulation of ESE-3b levels was induced by different TLR ligands as well as under pro-inflammatory conditions using the Jonuleit cytokine cocktail, it seems that upregulation of ESE-3b expression in immunogenic DC is not restricted to a certain maturation stimulus. Our findings are in agreement with previous reports showing induction of ESE-3 expression by pro-inflammatory cytokines like IL-1β and TNF-α in epithelial cells [27], [47]. Also the downregulation of ESE-3b in tolerogenic moDC populations was independent of the compound used to generate the cells. In accordance with our previous findings where we showed that reduction of ESE-3 during generation of moDC using siRNA leads to an impaired phenotype with remaining expression of CD14 and reduced expression of CD1a, HLA-DR and CD86 [15], the tolerogenic moDC populations analyzed in the present study showed remaining CD14 expression and reduced CD1a and HLA-DR surface expression (Figure 3) with low amounts of ESE-3b present. In all moDC population ESE-3a was expressed at much lower levels than ESE-3b (Figure 4B). In contrast to ESE-3b, ESE-3a mRNA levels were similar in immature and immunogenic moDC (Figure 5A). These results suggest that ESE-3a has not the same important role in development of immunogenic moDC as ESE-3b.

In general, the amount of mRNA does not necessarily correlate with the amount of protein as there are multiple possibilities of posttranscriptional regulations. Concerning ESE-3, previous studies using prostate cancer cells did not always reveal a direct correlation between ESE-3 mRNA levels and cellular protein content [31], [32]. In our experiments, upregulation of ESE-3b mRNA was accompanied by a high protein-level of ESE-3b. Both, at mRNA and protein level, ESE-3b followed the same expression pattern in the different moDC populations (Figure 5B, C). This suggests that ESE-3 gene expression rather than post-transcriptional regulation is responsible for the availability of ESE-3b protein in the nucleus. ESE-3a was not detectable at protein level, probably due to the lower sensitivity of Western blotting compared to quantitative real-time RT-PCR.

The third isoform described, ESE-3j, could not even be detected at mRNA level in any of the analyzed DC populations. Since the existence of ESE-3j has not been experimentally confirmed yet, further analyses are needed to verify the actual existence of this proposed ESE-3 isoform.

In order to establish the biological relevance of ESE-3 expression in DC, we extended our analyses to three DC populations directly isolated from peripheral blood. ESE-3b mRNA was detectable in mDC1, mDC2 and pDC (Figure 6) whereas ESE-3a was not detectable in any blood DC population. However, all blood DC populations had lower ESE-3b mRNA levels compared to immature moDC indicated by higher delta Ct values.

Our findings suggest that ESE-3b is the functionally most important ESE-3 isoform in DC making this an exciting target to enhance the immunostimulatory capacity of moDC used in immunotherapy.
